# A case of zootherapy with the tarantula *Brachypelma vagans *Ausserer, 1875 in traditional medicine of the Chol Mayan ethnic group in Mexico

**DOI:** 10.1186/1746-4269-7-12

**Published:** 2011-03-30

**Authors:** Salima Machkour-M'Rabet, Yann Hénaut, Peter Winterton, Roberto Rojo

**Affiliations:** 1 Laboratorio de Bioconservación ante el Cambio Global, El Colegio de la Frontera Sur (ECOSUR). Av. del Centenario Km. 5.5, C.P. 77900, Chetumal, Quintana Roo, Mexico; 2 Université Paul Sabatier Toulouse III, 118 route de Narbonne, 31062 Toulouse cedex, France

## Abstract

**Background:**

In practically every human culture, the use of arthropods as medicinal resources has been reported. In Mexico, the Mayan people mainly use plants but occasionally also animals and minerals in their medicine. This article is the first to report the traditional use of the tarantula *Brachypelma vagans *by medicine men in the Chol community, an ancient indigenous group that inhabits the southeastern part of Mexico. We also describe the utility of such arachnids in traditional medicine.

**Methods:**

This study was carried out in different Chol communities in the states of Chiapas and Campeche (southeastern Mexico) from 2003 until 2007. We interviewed the local medicine men, patients and non-Chol people in each village visited to collect information about the rituals involved and the effectiveness of this traditional medicine and also their opinion of this traditional medicine.

**Results:**

In all independent villages, the people who present an illness called 'aire de tarantula' or tarantula wind with symptoms including chest pain, coughing and asthma, were treated by the medicine man (called 'hierbatero') with a tarantula-based beverage. From village to village, the beverage has a similar base composition but some variations occur in additional ingredients depending on the individual medicine man. Like in all traditional Mayan medicine, the ritual of the ceremony consists of drinking the tarantula-based beverage and this is principally accompanied by chants and burning of incense.

**Conclusions:**

The recipe of the tarantula-based beverage and the procedure of this ritual ceremony were fairly constant in all the villages visited. Our work shows that despite the tarantula's bad image in several cultures, in others positive use is made of these spiders, as in modern medicine.

## Background

The use of plants, animals, mineral substances and other natural materials in traditional medicine by indigenous peoples, throughout the world and across time, is a well documented practice. Although plants and plant-derived materials constitute the principal source of ingredients for traditional medicine, the identification of animal resources for medical cures is also important [1,2].

Animal-based medicines can be prepared from the entire animal, from parts of the animal's body, from products of its metabolism (body secretions and excrement), or from other materials related to animals (nests and cocoons) [3]. The practice that uses animal or animal-derived products in human healing is known as zootherapy [3], according to the zootherapeutic universality hypothesis [4], zootherapy is widespread across most human cultures [5-8]. Traditional medicines use animal or animal-derived products from all taxonomic groups like echinoderms, insects, arthropods, reptiles, birds and mammals [9,8,11]. For example, in Sudanese traditional medicine fresh manure of a dromedary (*Camelus dromedaries *L. 1758) is used to alleviate arthritis [6]; in Nigeria, the hippo tusks (*Hippopotamus amphibious *L. 1758) are used as an aphrodisiac, the fat extracted from a manatee (*Trichechus senegalensis *Link 1795) is used to cure rheumatism, boils and backache [5]; in China, earthworm extract is prescribed to treat over 80 diseases like asthma, hypertension, ulcers, and epilepsy among other things [12].

The use of arthropods in traditional medicine is also widespread. In Chhattisgarh (India) over 500 insects, mites and spiders have been reported as useful to medicine to cure common and complicated ailments. For example, the red velvet mite (*Trombidium grandissimum *Koch 1867) is commonly used for paralysis, the bed bug (*Cimex lectularius *L. 1758) is used in the treatment of epilepsy, piles, alopecia and urinary disorders [9]. In different ethnic communities of India, 22% of the animals reported in traditional medicine (of a total of 109) are invertebrates used for diseases like asthma, tuberculosis, coughs and colds [10]. In northeast Brazil, the use of insects is common in medicine (14% of the listed medicinal animals in this region) principally for asthma, pneumonia, sinusitis, and coughs [11]. In the region of Chiapas (Mexico), 12 insects (16% of the animals listed) are used in the traditional medicine of the Mayan communities to cure coughs, warts and stammering [8].

A common use of spiders by man, in addition to keeping tarantulas as pets, is their consumption as food. In Cambodia, it is traditional to eat fried tarantulas (*Haplopelma albostriatum *Simon 1886: Aranea, Theraphosidae), and the Piaroa Indians of Amazon eat the Goliath bird-eating tarantula (*Theraphosa blondi *Latreille 1804) to become better hunters. The use of spiders in traditional medicine is very little documented. It was cited in India by Oudhia [9] and reported by Lev [13] with the use of crab spiders for healing in medieval times. Unfortunately, no details of spider species or their uses in traditional medicine are provided. A few other works report the use of spiders in medicine; in Brazil, Costa-Neto [14] reported the use of the chelicerates from the Goliath bird-eating tarantula to treat 'erysipelas' (or 'Holy fire'), fortification of teeth and asthma, and Costa-Neto and Resende [15] reported, in the city of Feira de Santana (Bahia State), the use of toasted bird-spider (referred to as mygalomorphs spp.) for sufferers of asthma; in Chiapas, Enríquez Vázquez [8] mention that Tzotziles and Tzetzales ethnic groups use a 'big spider' in their medicine and, Hunn [16] describes the use of a tarantula also in Chiapas (Mexico) to treat tumors, the patient being bitten in the affected zone. In this case, we may suspect that the tarantula in question is the Mexican redrump tarantula (*Brachypelma vagans *Ausserer 1875), as it is the only one known to occur in this area [17].

The use of tarantulas throughout the world is often to treat asthma-like conditions as mentioned above. Various species of tarantulas were used to treat the diseases and many themselves provoke asthma following the inhalation of tarantula hairs [18,19]. The most likely hypothesis to explain the role of tarantula setae in the asthma reaction is the action of the chitin particles [for more details of the mechanism of action see: 20,19].

### Mexican redrump tarantula

The genus *Brachypelma *(Mygalomorphae: Theraphosidae) comprises 21 species, registered in the CITES data base; among them, 14 occur in Mexico. The limited geographic distribution of this genus, the destruction of its habitat by landscape fragmentation, the high mortality rates in juveniles, the late age of sexual maturity (7-8 years for males, 9-10 years for females), and their high value in the pet trade, make all species of *Brachypelma *endangered [21,22] and therefore listed in appendix II of CITES since 1995.

*Brachypelma vagans*, commonly known as the Mexican redrump tarantula, has the widest distribution, being reported in the South of Mexico, Belize, El Salvador, Guatemala, Honduras and Costa Rica [17]. However, it has also been recorded in the wild in Florida [23], as trade has promoted its dispersion out of its natural range. This species is big, conspicuous and very abundant in some parts of the study area [24]. Recent ecological studies [24,25] show the close relation between the presence of *B. vagans *tarantulas and the traditional Mayan villages in the peninsula of Yucatan (Mexico). This relation is also common in Chiapas (Mexico) under similar conditions (unpublished data). The tarantulas are present and numerous in the center of the villages, in an open place used as a football or volley ball field and particularly for school activities, and in the backyards of the surrounding houses with 0.02 to 0.1 individuals per square meter [24,25]. A genetic study indicated that the Mexican redrump tarantulas occur in populations with a high number of related individuals. Each population is also genetically characteristic of each village where they are found [26]. These results seem, to confirm the probable relationship between the human populations of this region and the presence of *B. vagans*.

### Chol community

The Choles are indigenous peoples living in southeastern Mexico, mainly in the highland of Chiapas (named 'Los Altos'). After their rebellion (18^th ^century), with other Mayan ethnic groups (Tzoltziles and Tzeltales) in opposition to the Spanish colonial invasion, the Chol community settled mainly at the borders of the rainforest in Palenque, Tila, Tumbalá and Bachajón in Chiapas and Retalhuleu in Guatemala. Now, the two major communities of Chol are in Tila and Tumbalá.

The main economic activity is agriculture especially corn and beans ('frijol'), as well as sugar cane, rice, coffee, and various fruits. The Choles call themselves 'Winik', a Mayan word that means 'man or male'. They are the 'milperos' which refers to the people whose lives and existence have revolved around the cultivation of maize, their most sacred food. The Chol culture is very rich in traditional medicine. The medicine man, or 'hierbatero', plays a fundamental role in the community, not only as a doctor but also as a friend, psychologist, confessor, and re-establisher [27].

In the unusual 1921 Mexican census, residents of each state were asked to classify themselves in several categories, including pure indigenous, indigenous mixed with white and white. Chiapas State had a total of 98105 persons (five years of age and older) speaking at least twenty-five indigenous languages, representing 27.4% of the over 5 state population. The Chol language was spoken by 10.5% (10330 speakers) of those using indigenous languages in 1921. According to the 2000 census, the population of persons, five years old and more, who spoke indigenous languages amounted to 809592 individuals in Chiapas of which the Chol language represented 17.4% (104806 speakers) [28].

## Methods

### Study area

**Figure 1:**
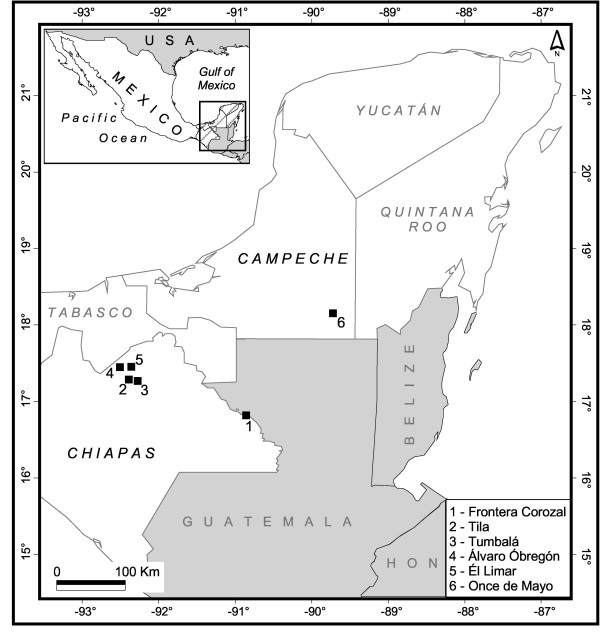
**Locations of the Chol communities visited for this study where the tarantula is used in traditional Mayan medicine (southern Mexico)**.

The study area was located in the states of Chiapas and Campeche (southern Mexico) (Figure 1). We investigated a total of six sites; five communities are in Chiapas: 1) Frontera Corozal (16°49'N, 90°53'W; altitude 115 m ) located on the banks of the Usumacinta river that marks the border between Mexico and Guatemala and is inhabited mainly by Chol people who arrived at least 30 years ago, 2) Tila (17°17'N, 92°25'W; altitude 1063 m ) is the major concentration of the Chol community and is a major religious centre, it is also inhabited by many mestizos who speak Chol language. It is an old town in Mexico that dates from the beginning of the colonial period, 3) Tumbalá (17°16'N, 92°18W; altitude 1490 m) contains a very large Chol population, 4) Álvaro Obregón (17°26'N, 92°32'W; altitude 390 m ) is a little Chol community close to Tila village, and 5) El Limar (17°27'N, 92°23'W; altitude 80 m) contains a large Chol population; the sixth community is in Campeche: 6) Once de Mayo (18°09N, 89°45'W; altitude 267 m) is a small village of around 400 inhabitants, located on the edge of the Calakmul Biosphere Reserve where we encounter Tzeltal, mestizo and Chol peoples.

### Data collection

**Figure 2:**
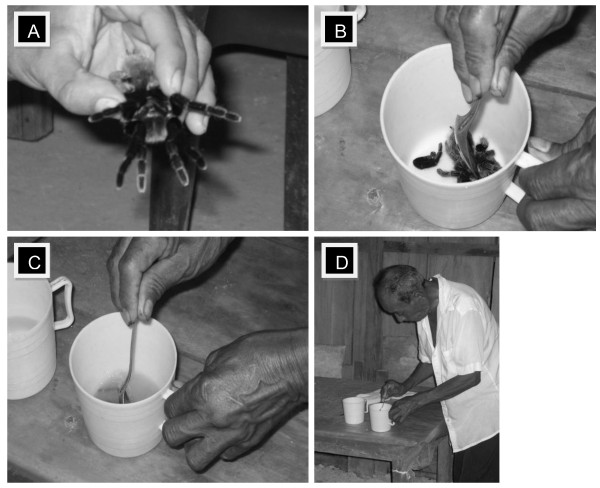
**Representation of the ritual to prepare the tarantula-based beverage used in traditional Mayan medicine in Mexico**. A) Medicine man kills the tarantula; B) Crushes it; C) Mixes it with spirit alcohol and some additional herbs; D) Filters the beverage to eliminate fragments and urticating hairs.

In order to understand the ritual of the use of the tarantula in traditional Chol medicine, we interviewed the medicine man, patients and some other target people in the six villages. To interview the medicine man in each of the villages we established a questionnaire to understand the practice of tarantula use (Table 1). We obtained the authorization to take a few photos during the ritual (Figure 2). The interview with the patients consisted in asking them about how they felt, before and after visiting the medicine man and their symptoms. The interviews of the village people included non-Chol people living in a mixed but mainly Chol community, to record their opinion about traditional practices in Chol medicine.

**Table 1:** Synthesis of the interview with the six (two in "Once de Mayo village) medicine men in five (excluding El Limar village) Chol traditional villages in Chiapas and Campeche States of Mexico

Answers	Reponses
Take pulse	Yes: 83% (n = 5); Unknown:17% (n = 1)
Use additional plants	Garlic + Tobacco + Caraway: 67% (n = 4) Garlic + Tobacco + pepper: 16.5% (n = 1) Garlic + Tobacco: 16.5% (n = 1)
Mixed with alcohol	Yes: 83% (n = 5) (one used hot alcohol) No: 17% (n = 1) (replaced by Holy water)
Ritual singing	Yes: 67% (n = 4); No: 16.5% (n = 1); Unknown: 16.5% (n = 1)
Ritual prayer	Yes: 100% (n = 6)
Patient drinks beverage	Yes: 83% (n = 5); No: 17% (n = 1)
Medicine man drinks beverage	Yes: 50% (n = 3); No: 50% (n = 3)
Spit in the face	Yes: 33% (n = 2); No: 67% (n = 4)
Burn the spider	Yes: 83% (n = 5); No: 17% (n = 1)
Use the ashes	Yes: 83% (n = 5); No: 17% (n = 1)
How many consultations	Two or three times: 67% (n = 4); 8 times: 17% (n = 1) Until the patient is cured: 17% (n = 1)
Payment	Chickens: 83% (n = 5); Other food: 17% (n = 1)

## Results

The use of *B. vagans *was prescribed for the same symptoms in all the Chol villages and villages with a mix of Chol and people of various other origins.

The patient comes to the 'hierbatero' (or herbalist) who takes the pulse at the wrist while he asks about the patient's illness and also his/her relational problems with people from the community. This first contact could be in the presence of some of the patient's relatives, to understand the relation with the good or evil powers. Once the medicine man has discarded other sicknesses like 'espanto' (fright; called 'susto' that symptoms comprise chest pressure, high blood pressure, chill, difficult to breath, general pain of legs and arms and constant fright), and reaches the conclusion that the patient is suffering from 'aire de tarantula', he asks the patient for a tarantula for the next session or gets both the animal and the necessary herbs himself. One of the 'hierbateros' explained: "This evil comes when a tarantula is hanging on the heart and eating it". The different names used in the Mayan vocabulary (or more precisely Chol vocabulary) to name spiders ('Chiwoj', 'Chiboj' [29]; or 'Chiuó') seem to be specifically used to designate the tarantula. In the Chol communities, the word 'Chiwoj' is commonly used for any tarantula-like spider but they use the name especially for *Brachypelma vagans*, the most conspicuous and biggest species of tarantula in the area.

The first examination by the 'hierbatero' determines the symptoms of 'aire de tarantula'. The symptoms presented by the patients diagnosed as suffering from the condition and those mentioned by the 'hierbateros' in all sites, are concurrent and are of respiratory nature like chest pain and coughing. In Tila village, one 'hierbatero's' wife mentioned that 'aire de tarantula' is what modern doctors call asthma.

The day after the first visit, ingredients are prepared and when the patient comes for his second appointment the 'hierbatero' lights incense, sits in front of the patient and begins to pray asking the Catholic god for the health of his patient and proceeds to kill the spider by pressing the anterior central part of the body (prosoma) longitudinally, between legs II and III with the forefinger and the thumb. Even if the spider is still moving, he puts it into a mug and mashes it with a spoon before adding 96° spirit alcohol, filling the goblet ¾ full. He then generally adds tobacco (*Nicotiana *sp. L. 1754), garlic (*Allium sativum *L.), caraway (*Carum carvi *L.) and sometimes other plants. The contents are mixed and filtered three times with a traditional Mayan cloth (called 'paliacate').

When the 'hierbatero' has finished preparing the tarantula-based beverage, he sits in front of the patient, starts to pray, sanctifies the beverage (the Chol people have a mixed religion between Catholicism and their ancient beliefs), and then blows in the patient's face spraying the liquid. He usually drinks a little too. The patient, who until now has been motionless, has to drink all the remaining liquid and then blows it in the 'hierbatero's' face.

According to some reports, the 'hierbatero' generally puts the remains of the spider in a frying-pan until they are reduced to ash. The use of the ash is very variable: 1) it can be added to the tarantula-based beverage and drunk; 2) smeared on the patient's skin, sometimes making a cross form on the chest or on the back or; 3) tied tightly to the patient's skin with cloth.

Depending on the strength of the illness they need to visit the medicine man two or three times and when the treatment has finished, they generally pay him with hens but sometimes with food or other items.

The 'hierbateros' proceeded in almost the same way with some slight differences like the use of some herbs and the disposal of the tarantula ashes.

The community of El Limar was the only one where the 'hierbatero' did not use the tarantula to cure this illness. He used the tarantula to call the soul of the patient suffering from a condition called 'espanto' (fright) [30]. This can occur when the soul has been caught by the Earth powers like caves and rivers, for instance. To cure 'espanto', the 'hierbatero' uses a mixture of spiritual treatments and herbs (praying, offerings and threats). In this case, they put the entire tarantula in a jar with spirit and camphor, and then let the mixture marinate for a couple of days or sometimes the 'hierbatero' prepares the mixture in advance.

According to testimony, the patients are cured. People who went to see the 'hierbatero' were very sick and after treatment felt well.

Only two patients were found in the village Once de Mayo. The symptoms of both patients were pain in the chest (lungs), difficult breathing and dry cough. The patients visited the medicine men two to four times, and both were totally cured.

The opinion of most of the non-Chol people is negative; they reject this traditional medicine. However, one of the patients who was non-Chol, but was from Veracruz State, said that the tarantula-based beverage helped to cure him.

One of us (R.R.) tasted the beverage and contrary to what could be thought, did not feel any irritation in the throat which could be expected because of the urticating hairs, classified as true setae [19], many of which are likely to have passed through the rough cloth sieve.

## Discussion

Many specialists consider spider danger to be over-evaluated [31] and that only few species are really dangerous for humans [32]. In the study area, *Brachypelma vagans *is not considered as a dangerous animal, and is found in abundance within villages [24,25]. It is well accepted by the people who do not attempt to eradicate them and on the contrary use them as a medicinal resource.

Generally spiders, and especially tarantulas are thought to be dangerous, as observed in traditional zoological knowledge in Brazil where a relationship was established between traditional and academic knowledge [14]. Also, the danger of spider bites is mentioned in academic work [18]. Other works mention eye diseases caused by the urticating hairs of tarantulas kept as pets [33,34]. The bad reputation of tarantulas not only stems from the possibility of them causing human disease but also from animal-lore. In Chiapas (Mexico), as in many other parts of Central and South America, there is a widespread belief, that the tarantula bites horses which can provoke fall of the hoof and even rotten leg leading the death of the animal. For this, the tarantula has other names like 'hierba' (grass), 'mala hierba' (weed), 'mata caballo' (horse killer) or 'pica caballo' (horse stinger) [35]. This 'attack' of horses by the tarantula is also related by Chol people.

Sometimes, the Choles mention the story but it was just as an anecdote related to the spider but of a very different nature from the 'aire de tarantula'. In El Limar village, there were more stories about mortal wounds to the hoofs of livestock than in the other villages in the study. The reason could be, like the fact that they do not use the tarantulas to cure the 'aire de tarantula', a result of the transculturalization brought about by the recently opened routes of communication. Besides that, El Limar is under the influence of the neighboring town of Salto del Agua, a very important fluvial dock including a railway that goes from Merida City, in the north of the Yucatan peninsula, to Mexico City. This encourages a mixture of cultures from southeastern Mexico, although it is still a Chol region.

This study revealed the similarity of the ritual associated to the use of the tarantula in traditional Chol medicine in all places investigated. In nearly all villages, the tarantula is crushed and mixed with alcohol and additional plants, and the beverage is filtered to eliminate pieces and urticating hairs. We assume that the effectiveness of the cure must come from one of the ingredients of the beverage. The ritual involves reciting prayers and singing by the medicine man. Then, the patient can drink the beverage. The similarity of protocol between very distant villages suggests that the use of the tarantula in traditional medicine is ancestral and well distributed throughout the area. Probably, this traditional use of *B. vagans *by the people contributed to maintaining the local population of this endangered tarantula.

We did not observe a marked difference in opinion about the use of this traditional ritual among the Chol and non-Chol peoples; both of whom use the services of the 'hierbatero'. However, some non-Chol people in mixed villages as in 11 de Mayo, criticize the 'hierbatero' as someone who in their opinion, exploits, people's ignorance to make money.

Tarantulas have been very well studied for the benefits they provide to medicine and particularly to pharmacology research like the case of the venom from the tarantula *Grammostola spatulata *Walckenaer 1837 (synonym *G. rosea *known as Chilean rose tarantula) now used as a drug to inhibit atrial fibrillation [36]. The venom peptide (known as GsMtx-4) works directly blocking the excitatory (action on stretch-activated ion channels; SACs) currents responsible for arrhythmia [36,37]. The use of GsMtx-4 as a drug, opens new clinical horizons in the diagnosis and treatment of pathologies including cardiac arrhythmia, muscular dystrophy and glioma [38,39].

Our study of this cultural tradition is a first approach to the components of the tarantula *Brachypelma vagans *body (including venom, urticating hairs, hemolymph and other tissues) to determine the beneficial and active substances that this tarantula may offers to pharmacology and hence to modern medicine.

## Conclusions

Our study clearly shows the common use of a spider (*Brachyplema vagans*) in traditional Mayan medicine throughout the regions of Mexico in which the Chol communities live. Furthermore, this is the first time that the whole ritual is reported in detail. Spiders, considered negatively in most cultures, are used positively in the Mayan culture, as they are in modern medical research.

## Competing interests

The authors declare that they have no competing interests.

## Authors' contributions

SMM conceived the study, participated in its design and draft final version of the manuscript. YH conceived the study, participated in its design and coordination and draft final version of the manuscript. PW participated in the design and draft of the manuscript. RR realized filed observations and draft first version of the manuscript. All authors read and approved the final manuscript.
